# Invisible victims: rising pediatric cocaine exposures in France (2020–2024) – insights from the national poison center database

**DOI:** 10.3389/ftox.2026.1732108

**Published:** 2026-02-25

**Authors:** Katharina von Fabeck, Mathieu Glaizal, Corinne Schmitt, Romain Torrents, Nicolas Simon

**Affiliations:** 1 Department of Clinical Pharmacology, Aix Marseille University, APHM, Institut de Neurosciences de la Timone, Marseille, France; 2 APHM, Hop Sainte Marguerite, Service de Pharmacologie Clinique, CAP-TV, Marseille, France

**Keywords:** blood analysis, breastfeeding, children, cocaine, intoxication

## Abstract

**Background:**

Cocaine use remains prevalent in Europe and has been associated with pediatric exposures through accidental ingestion, passive inhalation, and perinatal or postnatal transmission, potentially leading to significant toxicity in young children.

**Objective:**

This study aimed to quantify the number of pediatric cocaine exposures for which consultation with a French poison center was requested, characterize clinical presentations and severity, evaluate medical interventions and outcomes, and assess child protection service involvement.

**Methods:**

We conducted a retrospective observational study of children aged 0–10 years with suspected or confirmed cocaine exposure reported to French Poison Centers from 1 January 2020, to 31 December 2024. Data collected included demographics, exposure route, clinical manifestations, toxicological analyses, treatments, outcomes, and Poisoning Severity Score (PSS).

**Results:**

A total of 113 suspected pediatric exposures were identified, of which 76 (67%) were confirmed by toxicological analysis. Median age was 1.8 years, and 63 children were younger than 3 years. Exposure routes included intrauterine exposure (n = 7), breastfeeding (n = 12), ingestion (n = 9), and inhalation (n = 1). Most cases were symptomatic, with 25 minor (PSS 1), 24 moderate (PSS 2), and 8 severe cases (PSS 3), one fatality (PSS 4). No consistent association between measured cocaine or metabolite concentrations and clinical severity was observed in the limited number of cases with quantitative data (n = 15). Supportive care was sufficient in most cases, while 17 children required specific medical interventions.

**Conclusion:**

Pediatric cocaine exposures represent a significant clinical and public health concern, occurring through multiple pathways without predictable dose-response relationships. Clinical assessment must be guided by physical examination rather than quantitative toxicology alone. Prevention efforts must target households with substance use disorders.

## Introduction

In recent years, cocaine consumption has remained high in Europe and France, with measurable increases in lifetime and annual use among adults. According to international drug monitoring data, millions of adults in Europe report lifetime cocaine use, and prevalence estimates among young adults (15–34 years) vary by country but average several percent of the population (European Monitoring Centre for Drugs and Drug Addicti on, 2021; UNODC, 2021; OFDT, 2023). Europe accounts for one of the largest regional markets for cocaine worldwide, with millions of annual users and substantial levels of seizures reflecting widespread availability and use. In France, the most recent national drug monitoring reports indicate that cocaine is the second most prevalent illicit drug after cannabis, with approximately 9.4% of adults (18–64 years) reporting lifetime use and an estimated 1.1 million individuals reporting use in the past 12 months in 2023, roughly doubling earlier annual prevalence estimates from previous years (European Monitoring Centre for Drugs and Drug Addicti on, 2021). These data suggest widespread and persistent cocaine use across diverse demographic groups.

Such consumption patterns have important public health implications, including for children who may be unintentionally exposed. Pediatric exposure to cocaine can occur through multiple routes. Accidental ingestion of cocaine or crack residues left in domestic environments is a recognized risk, particularly for toddlers and infants. Passive inhalation of volatilized cocaine in households where smoking occurs may also lead to significant exposure. Prenatal exposure due to maternal drug use during pregnancy and postnatal exposure through breastfeeding remain critical vectors of early-life contact with cocaine. Depending on the dose and route of exposure, children may develop a wide range of clinical manifestations, including cardiovascular symptoms (e.g., tachycardia, hypertension), neurological complications (e.g., seizures, irritability), and in severe cases, life-threatening toxicity.

Despite the documented prevalence of cocaine use in adult populations, data describing pediatric exposures remain limited. National poison center-based analyses provide valuable insight into exposure circumstances, clinical severity, and management, but such studies are scarce (Gummin et al., 2024; NPIS, 2024). The aim of this study was to quantify the number of pediatric cocaine exposures for which consultation with a French poison center was requested, characterize the clinical presentation and symptom severity of intoxicated children, evaluate the medical interventions required including hospital management and outcomes, assess the involvement of child protection services, and describe related social health considerations.

## Methods

This retrospective observational study analyzed pediatric cases of suspected or confirmed cocaine exposure reported to the French Poison Centers between 1 January 2020, and 31 December 2024. Eligible cases included all patients aged 0–10 years with cocaine or cocaine derivatives (e.g., crack cocaine) coded as a suspected or confirmed agent in the French National Poison Center Database (SICAP), regardless of the route of exposure (oral, inhalational, transplacental, or via breast milk). Case identification was based on information reported by healthcare professionals or caregivers at the time of consultation, with or without toxicological confirmation. Extracted variables included demographic characteristics, exposure circumstances, clinical presentation, laboratory and toxicological findings, and treatment modalities, including decontamination, pharmacological interventions, and intensive care unit admission.

Severity of poisoning was classified using the Poisoning Severity Score (PSS) (Persson et al., 1998), and outcomes were assessed at discharge and at follow-up (when available).

Confirmed cocaine exposure was defined as a case with a positive analytical toxicology result in urine screening performed by hospital-affiliated clinical or forensic laboratories.

### Clinical and toxicological workflow

All toxicological analyses were performed as part of routine clinical care in hospital or forensic laboratories involved in patient management; French Poison Centers do not carry out biological analyses or drug level quantification. First-line toxicology screening relied on qualitative urinary immunoassays routinely available in emergency departments or hospital laboratories, targeting benzoylecgonine (cocaine metabolite), cannabinoids, opioids, benzodiazepines, and amphetamines, and intended for initial clinical orientation only. Second-line confirmatory analyses using liquid chromatography–mass spectrometry (LC-MS or LC-MS/MS) were performed selectively, depending on clinical or judicial indication. Quantitative measurements of cocaine and its metabolites were therefore available only when specifically requested, explaining why concentrations were reported in only 15 of the 76 confirmed cases. Hair analysis by LC-MS/MS was used selectively in cases suggesting chronic, repeated, or prenatal exposure.

As this study was based on a national poison center database, toxicological analyses were conducted in multiple laboratories across France, each applying locally validated routine methods. Consequently, analytical sensitivity and limits of detection varied between laboratories and could not be standardized. Toxicological results were therefore primarily analyzed qualitatively (positive vs. negative), while available quantitative results were reported descriptively.

Routes of exposure were classified based on combined information from clinical history, emergency department reports, and child protection notes. Overlap between *in utero* and postnatal exposure (e.g., breastfeeding plus prenatal exposure) was considered possible and explicitly acknowledged when hair analyses suggested prenatal presence of cocaine.

### Symptom classification

Symptoms were categorized as relevant if they were compatible with sympathomimetic poisoning (tachycardia, hypertension, agitation, seizures, altered consciousness). Mild, non-specific symptoms (e.g., irritability, mild feeding difficulties) were included when explicitly documented. As documentation in retrospective datasets is limited, some underestimation of minor symptoms is possible.

### Statistical analysis

Analyses were primarily descriptive due to sample size and heterogeneity. Continuous variables are presented as medians; categorical data as frequencies. Temporal trends were described narratively without inferential testing. A Welch t-test compared PSS by sex; Pearson correlation assessed age vs. PSS. No multivariable modelling was performed, consistent with the exploratory nature of the dataset. All analyses were conducted using R (version 2025.09.0 + 387).

## Results

During the 5-year study period, 113 pediatric cases of suspected cocaine exposure were identified and reviewed. Only cases confirmed by positive urinary cocaine analysis (n = 76, 67.3%) were included in the final evaluation. In 15 cases, quantitative measurements of cocaine, norcocaine, benzoylecgonine, or ecgonine methyl ester were available in urine, blood, or hair.

**FIGURE 1:**
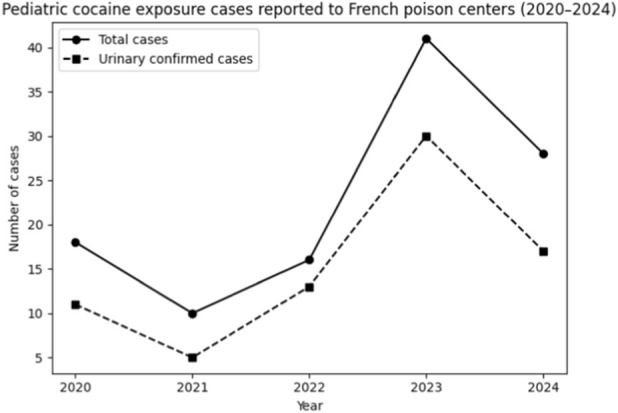
Number of cases based on year of intoxication.

The median age of the 76 children with confirmed positive urine screening results was 1.8 years (range: neonates to 10 years), with the majority being under 3 years of age (n = 63, 83%). The sex distribution was balanced (male:female = 37:39). Routes of exposure were often unclear; documented exposures included ingestion (n = 9, 11.8%), inhalation (n = 1, 1.4%), prenatal exposure (n = 7, 9.2%), and transmission via breastfeeding (n = 12, 15.8%). Parental drug use was reported in 32 cases (42.1%) (Table 1). Figure 1 provides a year-wise breakdown of the cases.

**TABLE 1:** Cocaine exposure in children by age group and associated factors.

Age group	0–1 month	1–3 months	3 months- 1 year	1–3 years	3–6 years	6–10 years	Total
Number of cases	12	4	19	28	6	7	76
Median age	0.038	0.191	0.689	1.858	3.906	7.021	1.828
Sex ratio m/f	6:6	2:2	11:8	12:16	2:4	4:3	37:39
Known route of exposure							
• Ingestion	​	​	​	8	1	​	9
• Inhalation	​	​	​	​	1	​	1
• Prenatal	7	​	​	​	​	​	7
• Breastfeeding	10	1	​	1	​	​	12
Known parental use	12	1	6	6	2	5	32
Poisoning severity score							
• PSS 0	6	1	2	7	2	0	18
• PSS 1	3	1	11	6	1	3	25
• PSS 2	1	1	5	12	2	3	24
• PSS 3	2	0	1	3	1	1	8
• PSS 4	0	1	0	0	0	0	1

Severity according to the Poisoning Severity Score (PSS) was as follows: 18 (23.7%) asymptomatic (PSS 0), 25 (32.9%) minor (PSS 1), 24 (31.6%) moderate (PSS 2), 8 (10.5%) severe (PSS 3), and 1 (1.4%) fatal case (PSS 4).

**FIGURE 2:**
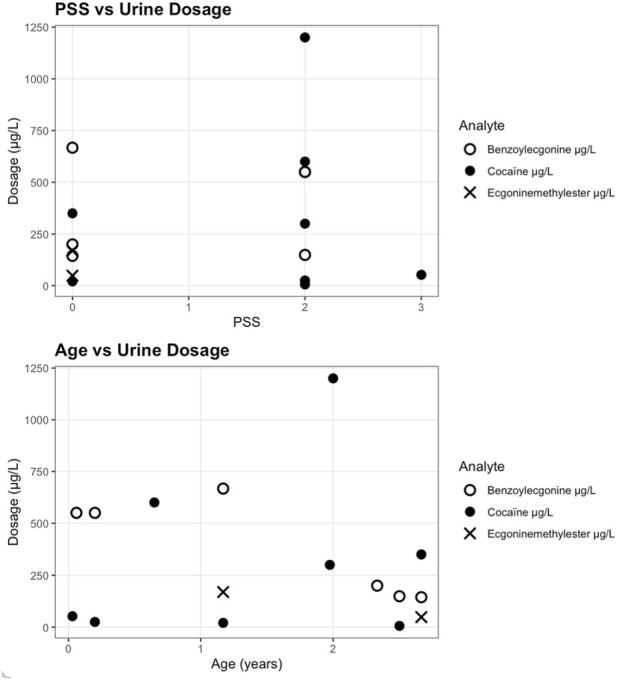
Urinary cocaine and metabolite concentrations by poisoning severity score (PSS) and age, n = 10.

**FIGURE 3:**
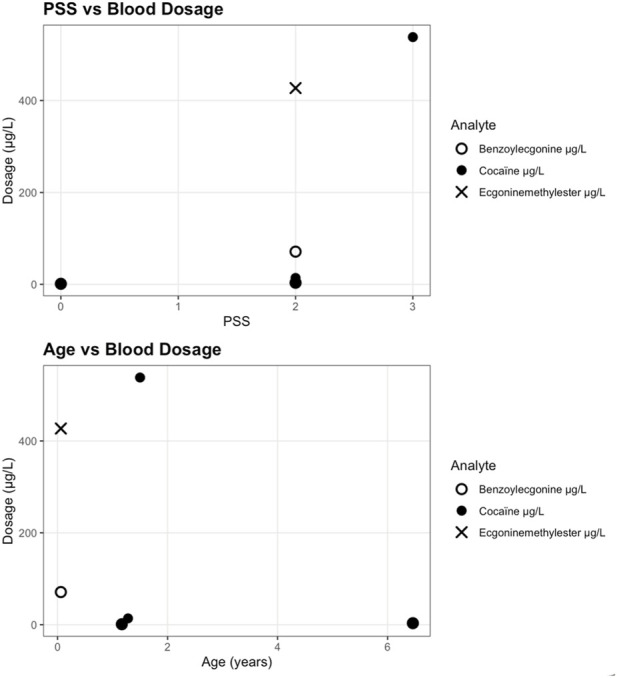
Blood cocaine and metabolite concentrations by poisoning severity score (PSS) and age, n = 5.

Quantitative analyses showed urinary cocaine concentrations ranging from 5.9 μg/L to 1,200 μg/L, benzoylecgonine from 144.4 μg/L to 667.1 μg/L, and ecgonine methyl ester from 48.2 μg/L to 169 μg/L. Serum cocaine levels varied from <2.5 μg/L to 538 μg/L, benzoylecgonine from 1.03 μg/L to 71.1 μg/L, and ecgonine methyl ester up to 427 μg/L. Hair analyses demonstrated a wide range of cocaine concentrations (0.187 ng/mg to 24.289 ng/mg). In the subgroup of 15 children with available quantitative analyses, scatter plots of urinary and blood concentrations (Figures 2, 3) did not reveal a discernible trend between measured levels of cocaine or metabolites and poisoning severity or age. Because only a minority of confirmed cases underwent quantitative testing (reflecting selective use of LC-MS/MS in clinical or medico-legal contexts) these data are insufficient to establish or exclude a dose-severity relationship. They do, however, illustrate substantial variability in measured concentrations across all clinical severity grades.

The Welch two-sample t-test revealed no significant difference in PSS scores between female (M = 1.26) and male (M = 1.41) participants, t (73.91) = −0.65, p = 0.52, 95% CI [-0.61, 0.31]. Similarly, Pearson’s correlation indicated no significant association between age and PSS scores, r = 0.15, t (74) = 1.32, p = 0.19, 95% CI [-0.08, 0.36]. These results suggest that poisoning severity score did not differ by sex and was not related to age in the current sample.

### Two illustrative severe cases (PSS 3) with quantitative analysis included

Case 1: An 11-day-old female infant, exclusively breastfed, presented with respiratory distress, muscular hypertonia, jaundice, and cardiac arrest upon emergency service arrival. Hair analysis revealed a cocaine concentration of 24.289 ng/mg; urinary cocaine was 52.5 μg/L, suggesting both prenatal and breastfeeding exposure. The patient required resuscitation at home. No other substances were detected in the blood screening.

Case 2: A 1.5-year-old girl with unknown route of exposure had a blood cocaine concentration of 538 μg/L. She presented with generalized tonic-clonic seizures, mydriasis, and hyperthermia (39.2 °C) refractory to midazolam, requiring treatment with diazepam, midazolam infusion, and oxygen. Aside from the positive cocaine test, she also showed a slight positive result for cannabis.

One fatal case (PSS 4) involved a 3-month-old male infant found dead at home; *postmortem* blood analysis confirmed the presence of cocaine. The child was found dead in his bed after breastfeeding from his mother, a cocaine user. While the cocaine levels were considered too low to have caused the death, the parents refused the autopsy. Toxicological screening revealed no other substances.

Of note, several children with urinary cocaine concentrations up to 300 μg/L remained asymptomatic.

Nine children, categorized into the following age groups: three aged 0–1 month, four aged 3 months to 1 year, one aged 1–3 years, and one aged 6–10 years, were removed from their biological family and placed into a different household. This action followed multidisciplinary assessment indicating unsafe home environments, recurrent drug exposure risk, or inconsistencies in caregiver accounts.

Toxicological screening for various substances revealed the presence of cannabis, THC, nitrous oxygen and opioids in the samples of 14 children. Co-exposure to cannabis resulted in at least a PSS of 1 (with agitation, somnolence or seizures), while co-exposure to heroin, methadone, and opioids did not lead to an increase in PSS (0). The child, exposed to recreational nitrous oxide during pregnancy, was born with mild facial dysmorphia, macrocytosis, and a markedly elevated blood homocysteine concentration. During the first year, the child developed hypervigilance, delayed neurodevelopment, and social issues.

Treatment interventions were documented across all pediatric cocaine intoxication cases as follows: The majority of patients (n = 53, 69.7%) required only supportive care with simple monitoring, reflecting the predominance of mild to moderate presentations. Active medical interventions were necessary in 17 (22.4%) cases.

Fluid resuscitation was the most frequently employed active intervention, administered to 5 (6.6%) patients to address dehydration or hemodynamic instability. Oxygen supplementation was provided to 3 (3.9%) patients for respiratory support, while benzodiazepines were administered to 4 (5.3%) patients for seizure control or severe agitation management.

Single cases required more intensive interventions: one patient necessitated external cardiac massage for cardiac arrest, another received vitamin B12 supplementation (co-intoxication with nitrous oxygen), and one patient was treated with vitamin K. Additional isolated treatments included analgesic administration for pain management, antihistamine therapy, and in one case, induced vomiting was performed as a decontamination measure.

## Discussion

This 5-year retrospective analysis of pediatric cocaine exposures reported to French Poison Centers provides important clinical and epidemiological insight into an emerging public health concern. The identification of 113 suspected cases, of which 76 were toxicologically confirmed, reflects a measurable burden of cocaine exposure among young children. The observed increase between 2020 and 2024 parallels national surveillance data from the French Monitoring Centre for Drugs and Drug Addiction (OFDT) and European Monitoring Centre for Drugs and Drug Addiction (EMCDDA), which report rising cocaine availability, hospitalizations, and record seizures since 2019 (European Monitoring Centre for Drugs and Drug Addicti on, 2021; OFDT, 2023; Eiden et al., 2022). Similar upward trends have been described internationally. Data from the U.S. National Poison Data System (Gummin et al., 2024) and toxicology cohorts in the United Kingdom (NPIS, 2024) report increasing stimulant-related exposures in children under 5 years of age, suggesting that the pattern observed in our cohort likely reflects broader population-level changes in cocaine circulation rather than improved detection alone.

The predominance of cases in children under 3 years of age highlights developmental vulnerability and environmental exposure risk. Accidental ingestion remains the most frequently reported mechanism in international pediatric series; however, our cohort demonstrates substantial heterogeneity of exposure routes, including intrauterine transmission, breastfeeding, ingestion, and inhalation (Yang et al., 2025). This diversity underscores the complex epidemiology of cocaine exposure in early childhood. Cocaine and its metabolites readily cross the placenta and are excreted into breast milk. Case reports and small neonatal series have documented irritability, seizures, and cardiorespiratory instability in breastfed infants following maternal consumption, sometimes with delayed symptom onset (Chasnoff, 1989; Bauer et al., 2005; Youn et al., 1992). The identification of 12 breastfeeding-associated cases in our dataset suggests that lactational exposure may be underrecognized and warrants systematic screening and counseling in high-risk maternal populations.

Clinical severity in our cohort ranged from asymptomatic presentations to one fatal case. The distribution across Poisoning Severity Score categories is consistent with international reports indicating that pediatric cocaine exposure is associated with a higher proportion of moderate to severe outcomes compared with many other unintentional pediatric poisonings (Claudet et al., 2023; Armenian et al., 2017). In contrast to opioids, where respiratory depression predominates, cocaine intoxication produces a sympathomimetic toxidrome characterized by tachycardia, hypertension, hyperthermia, seizures, and dysrhythmias (Lavonas et al., 2023). Nine children in our cohort experienced severe manifestations, including status epilepticus, respiratory failure, and cardiac arrest. The fatal case in a 3-month-old infant with confirmed *postmortem* cocaine illustrates that life-threatening toxicity can occur in very young children even in the absence of extraordinarily high measured concentrations (Pinto et al., 2013; Riggs and Weibley, 1991; Lustbader et al., 1998).

Quantitative toxicological data were available in only a minority of confirmed cases and did not demonstrate a consistent concentration-severity relationship. This observation aligns with existing toxicology literature indicating that measured blood or urine concentrations of cocaine and benzoylecgonine correlate poorly with clinical severity, particularly in pediatric populations. Several mechanisms may explain this variability. Cocaine undergoes rapid metabolism through plasma and hepatic esterases, and the timing of sampling relative to exposure substantially influences measured concentrations. Neonates and young infants exhibit developmental differences in hepatic enzyme activity, renal clearance, plasma protein binding, and blood–brain barrier permeability, which may alter both pharmacokinetics and pharmacodynamics (Roque Bravo et al., 2022; Kearns et al., 2003). These physiological factors limit extrapolation from adult dose-response paradigms and reinforce the primacy of clinical assessment over isolated laboratory values in pediatric intoxication.

Hair analysis in selected cases provided evidence suggestive of chronic or prenatal exposure. Forensic toxicology studies have demonstrated that segmental hair analysis can assist in reconstructing exposure timelines in infants, although interpretation remains complex due to the possibility of external contamination in environments where cocaine is actively used. Overlapping exposure pathways are therefore likely in households with sustained substance use, and strict categorization into single routes may underestimate multifactorial exposure (Pragst and Balikova, 2006).

Co-intoxications were identified in 14 children and likely contributed to clinical heterogeneity. Experimental and clinical data suggest that cannabinoids may potentiate central nervous system depression, while opioids increase the risk of respiratory compromise. Even in the absence of observed respiratory depression in our cohort, the potential for synergistic toxicity must be considered. Polysubstance exposure complicates attribution of symptoms and may partially explain severe manifestations at relatively low cocaine concentrations or conversely mild presentations at higher measured levels.

From a systems perspective, pediatric cocaine exposure functions as a sentinel indicator of adult substance use patterns. Experiences from the North American opioid crisis demonstrate how shifts in adult drug epidemiology translate into pediatric harm through unintentional household exposure. The increasing circulation of cocaine in Europe may produce a comparable secondary burden among young children. Preventive strategies must therefore extend beyond acute management and integrate addiction treatment access, maternal counseling during pregnancy and lactation, harm-reduction interventions, and coordinated medico-legal response when child safety is compromised.

Several cases in this study raise medico-legal concerns. High cocaine concentrations in infants, breastfeeding-related exposures, repeated exposures, or detection of multiple substances are recognised red flags for unsafe caregiving environments or neglect. French legislation (Article L226-2-1, Code de l’Action Sociale et des Familles) mandates reporting suspected child endangerment, including drug exposure (Legifrance, 2013). In practice, poison centers collaborate with pediatricians and forensic units. Judicial investigations may be initiated when intoxication circumstances appear inconsistent or intentional administration is suspected. The removal of nine children reflects multidisciplinary assessment indicating a high risk of recurrent exposure. These findings are consistent with prior forensic literature (Meinhofer and Angleró-Díaz, 2019; LaBrenz et al., 2022) which highlights stimulant exposure as an indicator of hazardous household environments.

Taken together, our findings reinforce that pediatric cocaine intoxication is clinically heterogeneous, potentially severe, and frequently embedded in complex social contexts. Early recognition based on toxidrome identification, vigilant physiological monitoring, and multidisciplinary collaboration remain central to optimal management. Quantitative toxicology, while informative when available, should be interpreted as contextual data rather than a determinant of severity.

### Limitations

This study has several limitations that warrant consideration. First, the retrospective nature of the data collection may have resulted in incomplete documentation of clinical presentations and exposure circumstances, particularly regarding routes of exposure which remained unclear in a substantial proportion of cases. Second, only 15 of 76 confirmed cases had quantitative measurements of cocaine and metabolites available, limiting the depth of pharmacokinetic analysis. This is reflecting the fact that quantification is not routinely performed and is ordered selectively by hospital clinicians or judicial authorities. Poison Centers do not perform toxicology testing, which depends on the capabilities of the receiving hospital. This may introduce selection bias toward more severe or medico-legal cases. Third, the study population was restricted to cases reported to French Poison Centers, which may not capture all pediatric cocaine exposures, particularly those managed outside the hospital setting or not reported to toxicology services, thereby potentially underestimating the true incidence. Finally, co-exposure testing was not uniform across all cases, and the detection of co-intoxicants in only 14 children may underestimate the true prevalence of polydrug environments.

## Conclusion

This study confirms that pediatric cocaine exposures continue to represent a significant clinical and public health concern, occurring through multiple exposure pathways without a clear predominant route. Quantitative toxicology was available only in a small proportion of confirmed cases, and within this subset no consistent association between measured concentrations and clinical severity emerged. Although the limited number of quantified samples restricts interpretability, the broad variability in clinical presentations across exposure levels suggests that concentration data alone cannot reliably predict toxicity in young children. Comprehensive bedside assessment therefore remains central to clinical decision-making, particularly in infants who may present with subtle or non-specific symptoms. Future research should aim to elucidate factors underlying asymptomatic courses despite measurable exposure and to refine risk stratification in this vulnerable population.

## Data Availability

The original contributions presented in the study are included in the article/supplementary material, further inquiries can be directed to the corresponding author.
